# Development of the Nurses’ Attitudes Toward Incarcerated Patients Scale: A Psychometric Study

**DOI:** 10.1111/inr.70086

**Published:** 2025-08-28

**Authors:** Büşra Dağci‐Günal, Sultan Ayaz‐Alkaya, Neslihan Köse‐Kabakcioğlu, Adnan Kan

**Affiliations:** ^1^ Bandırma Onyedi Eylül University Faculty of Health Sciences Balıkesir Türkiye; ^2^ Gazi University Faculty of Nursing Ankara Türkiye; ^3^ Gazi University Gazi Faculty of Education Ankara Türkiye

**Keywords:** attitude, correctional, incarcerated patients, justice, prison, psychometric analysis, reliability, validity

## Abstract

**Aim:**

To develop an original scale to measure nurses’ attitudes toward incarcerated patients and assess its psychometric properties.

**Methods:**

This study employed a psychometric testing approach involving test–retest design. The sample size was determined based on the rule of selecting a sample 5 to 10 times the number of scale items. The study included 672 nurses. Data were collected using the Descriptive Information Form and the Nurses’ Attitudes Toward Incarcerated Patients Scale. Content validity was determined using the Lawshe technique. Construct validity was examined through exploratory (*n*1  =  336) and confirmatory factor analysis (*n*2  =  336). Internal consistency was assessed using Cronbach's alpha coefficient. Temporal stability was evaluated through the test–retest method conducted four weeks later (*n*  =  32).

**Results:**

The scale consists of 12 items and was categorized into three factors—discriminatory attitudes, emotional discomfort, and patient equality—accounting for 67.6% of the variance. Items with factor loadings between 0.69 and 0.85 supported the scale's validity. The fit indices from confirmatory factor analysis indicated an acceptable model fit. Internal consistency was confirmed with a Cronbach's alpha of 0.84. Test–retest reliability analysis showed moderate, significant correlations among subdimensions.

**Conclusion:**

The scale is valid and reliable in assessing nurses’ attitudes toward incarcerated patients. Future research should test its applicability across diverse cultural and geographical contexts to enhance generalizability.

**Implications for Nursing Policy:**

This study underscores the need for policies addressing nurses’ attitudes toward incarcerated patients. The developed scale may support the identification of biases and emotional discomfort, guiding targeted education and institutional interventions to promote equitable, ethical, and nonjudgmental healthcare practices.

## 1 Introduction

Individuals incarcerated in prisons and detention centers exhibit sociocultural and socioeconomic differences regarding their health‐related attitudes and behaviors (Aktaş 2021). Incarcerated and convicted are considered medically and legally vulnerable groups and, due to their status, may face challenges such as limited treatment options, lower standards of care, and violations of privacy (Fuller and Eves 2017). Moreover, incarcerated individuals experience significantly poorer physical and mental health conditions compared with the general population. The prevalence of chronic illnesses, such as type 2 diabetes, asthma, and HIV/AIDS, is markedly higher among this population, yet these conditions often remain inadequately treated (Curran et al. 2023). Furthermore, the prevalence of severe mental disorders, including depression and psychotic illnesses, is also significantly elevated in correctional settings compared with the general community (Emilian et al. 2025). Given the constraints they experience, incarcerated individuals are dependent on external factors to meet their health‐related needs and address their medical concerns. This dependency hinders their access to healthcare services, placing them in a distinctly disadvantaged position among other vulnerable populations (Aslantürk et al. 2017).

In many countries worldwide, incarcerated patients are entitled to access healthcare services when needed, as mandated by law. According to the United Nations Standard Minimum Rules for the Treatment of Prisoners (also known as the Mandela Rules) (2015), prisoners must have access to the same standard of health care available in the community and should receive health services free of charge without discrimination on the grounds of their legal status. For instance, in the United States, the Federal Bureau of Prisons (2024) employs a system that facilitates the referral of inmates to designated hospitals. When prisoners require emergency or advanced medical treatment, they can be transferred to external healthcare facilities. Similarly, countries such as Canada, the United Kingdom, Germany, and the Scandinavian nations provide advanced medical care to prisoners when necessary. In the United Kingdom, the National Health Service (2023) delivers both in‐prison and external healthcare services, ensuring that inmates receive specialized medical interventions through hospital referrals. These practices aim to uphold the right to health for incarcerated individuals belonging to disadvantaged groups and to guarantee their access to comprehensive medical services (Hutchings and Davies 2021; Saloner et al. 2022). However, in certain cases, incarcerated patients require treatment in specialized units such as intensive care, operating rooms, emergency departments, or maternity wards (Durkal and Üşenmez 2023). Consequently, the responsibility of providing care to these patients extends beyond nurses working in‐prison and correctional facility wards to include all nurses serving in various healthcare units (Brooks et al. 2022).

## 2 Background

Nursing education plays a critical role in shaping nurses' attitudes, particularly toward vulnerable or marginalized populations such as incarcerated patients. However, many nursing curricula still lack sufficient coverage of correctional health topics (So et al. 2023). This situation may lead to feelings of unpreparedness, negative attitudes, or professional ambiguity among nurses (Isaac Caro 2021; Sutherland et al. 2021).

Nurses working in correctional settings face complex professional, ethical, and emotional challenges, including feelings of isolation, moral distress, and role conflict (Lazzari et al. 2020; Sasso et al. 2018). Studies have shown that correctional nurses are at high risk for burnout, secondary traumatic stress, and compassion fatigue due to the intense emotional needs of the incarcerated population they serve (Hancock 2020; Bell et al. 2019; Wright 2020). Moreover, the need to balance patient confidentiality with institutional security requirements creates ethical dilemmas that further intensify professional tension (González‐Gálvez et al. 2018). On the other hand, hospital‐based nurses often have limited experience in providing care to incarcerated patients, which makes the process more complex, stressful, and challenging (Wirmando et al. 2021a).

Several barriers complicate nurses' ability to care for incarcerated patients, including personal safety concerns (Goshin et al. 2019), stigma and discrimination, ethical dilemmas, and uncertainties regarding institutional policies and professional roles (Wirmando et al. 2021b). A qualitative study in the literature revealed key challenges experienced by nurses, such as workplace distress, emotional conflicts, security concerns, difficulties in establishing therapeutic relationships, unnatural caregiving processes, and the involvement of law enforcement officers in patients’ treatment procedures (Wirmando et al. 2021a). These barriers and challenges directly influence nurses’ attitudes toward incarcerated patients, leading to variations in caregiving behaviors.

According to the Theory of Planned Behavior, attitude is one of the three fundamental components that shape behavior. Therefore, nurses’ attitudes toward incarcerated patients may play a determining role in their behavior toward these groups (Mulyani et al. 2021). In the literature, several scales have been developed to measure attitudes toward incarcerated individuals, such as the “Attitudes Toward Self‐Harming Prisoners Scale” (Garbutt and Casey 2015), the “Attitudes Toward Former Offenders Scale” (Batur and Akbaş 2023), the “Nurses’ Attitudes Toward Forensic Psychiatric Patients Scale” (Baysan‐Arabacı and Çam 2011), the “Perceptions of Offenders Scale” (Gönültaş et al. 2019), and the “Attitudes Toward Offenders Scale” (Pereyra and Moreno 2001). However, these instruments are not specific to nurses’ professional roles, ethical approaches, and patient care processes. This limitation prevents a comprehensive evaluation of factors such as ethical dilemmas, communication difficulties, institutional constraints, and safety concerns that nurses encounter when providing care to incarcerated patients. Given this gap, developing a specific scale to assess nurses’ attitudes toward incarcerated patients is a critical necessity for clinical practice. The proposed scale is expected to provide a scientific framework for evaluating nurses’ attitudes, facilitating the identification of the current situation and contributing to enhancing the quality of nursing care.

The aim of this study was to develop a novel and psychometrically valid scale to measure nurses’ attitudes toward incarcerated patients and assess its psychometric properties. Research questions were as follows:

‐ Is attitudes toward incarcerated patients scale a valid measurement tool for nurses?
‐ Is attitudes toward incarcerated patients scale a reliable measurement tool for nurses?

## 3 Methods

### 3.1 Study Design and Participants

This psychometric study involved test–retest design. The COSMIN Reporting Guideline was utilized to report the scale development and validation process (Gagnier et al. 2021). In psychometric analyses, it is recommended that the sample size be at least 5–10 times the total number of scale items to conduct factor analysis (DeVellis and Thorpe 2021). To reach a broader nursing population and establish a comprehensive dataset, the snowball sampling technique was employed (Leighton et al. 2021). The online survey, created using Google Forms, was sent to nurses working in public institutions via WhatsApp and social media, with participants being encouraged to share it with their colleagues. Overall, 728 nurses were invited to participate in the study.

Of the recruited nurses (*n* = 728), 27 did not provide informed consent, and 29 inappropriately completed the instruments. The study was completed with 672 nurses, with 336 nurses assigned to the exploratory factor analysis (EFA) group and 336 nurses to the confirmatory factor analysis (CFA) group for the 35‐item scale.

For the test–retest reliability analysis, a sample size of 30 participants is considered sufficient for obtaining reliable results, as recommended by Tabachnick and Fidell (2020). Accordingly, the test–retest procedure was conducted with 32 nurses. To match participants’ responses, nurses were asked to use anonymous pseudonyms when completing the surveys. The second administration of the scale was conducted four weeks after the initial application with the same 32 nurses.

The inclusion criteria for the study were as follows: (1) being employed as a nurse in a public institution, (2) having the technical capacity to complete the online survey, and (3) having Turkish as their native language. The exclusion criteria included participation in the pilot study and employment in private healthcare institutions. In Türkiye, in accordance with legal regulations, incarcerated patients in need of healthcare services are referred to public institutions; therefore, nurses working in private healthcare facilities were excluded from the study. To ensure adherence to this criterion, the online survey form included a mandatory confirmation button stating, “I confirm that I am employed in a healthcare institution affiliated with a public organization.”

### 3.2 Scale Development Process

**FIGURE 1:**
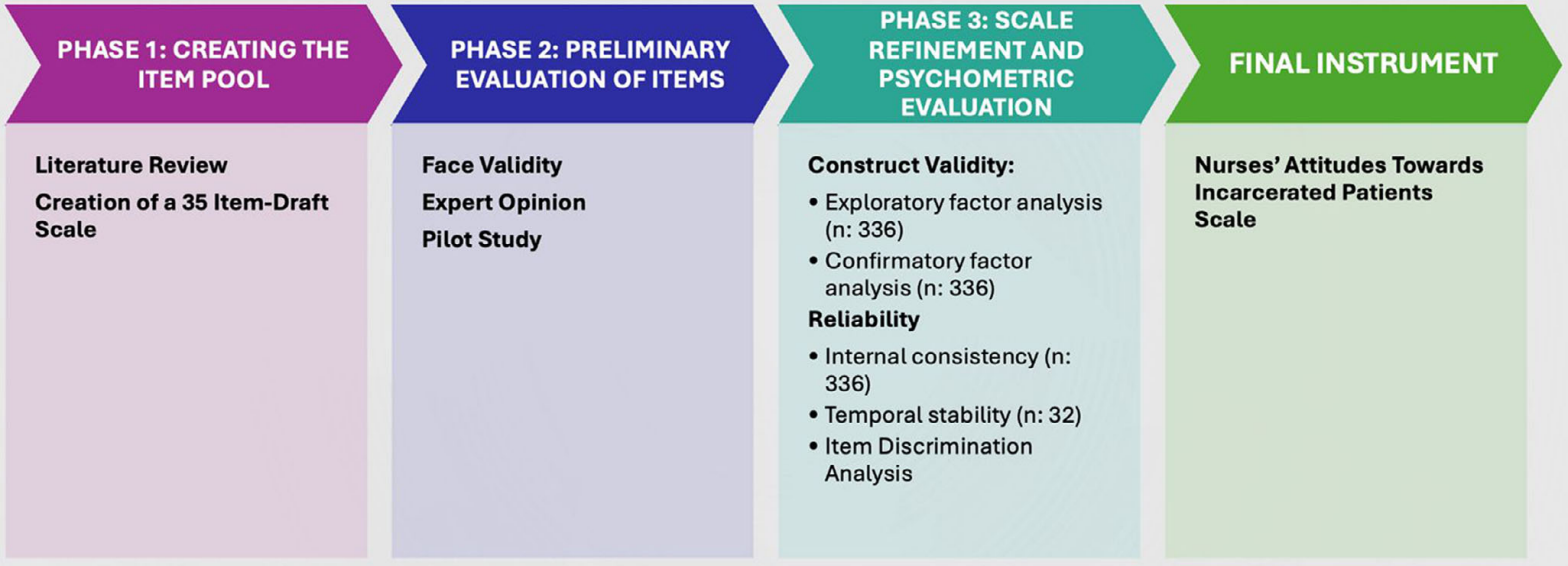
Development process of the nurses’ attitudes toward incarcerated patients scale.

The scale development process consists of three phases: (1) item pool creation, (2) preliminary evaluation of items, and (3) scale refinement and psychometric evaluation (Figure 1). In the first phase, a comprehensive literature review was conducted, and an item pool was generated. In the second phase, scale items were determined based on expert opinions and a pilot study. In the final phase, the scale was administered to the designated sample group, and analyses were conducted to assess construct validity, content validity, and internal consistency.

#### 3.2.1 Creating the Item Pool

To generate scale items, a literature review was conducted in Scopus, Medline, WOS, CINAHL, and Science Direct databases on nursing care for incarcerated patients. During item development, researchers considered the theoretical structure of attitudes (e.g., dimensions of attitude: cognitive, affective, and behavioral; intensity and strength of attitude) and elements aligned with this framework (e.g., wording, content, and intensity of attitude statements).

Following final evaluations with the authors, all items were reviewed, and ambiguous, double‐barreled, grammatically incorrect, or poorly structured items were revised. As a result, a final version of the scale consisting of 35 items was developed.

#### 3.2.2 Obtaining Expert Opinion

Expert opinions were obtained from 10 specialists (5 nurses and 5 nursing faculty members) to assess the developed structure. The Lawshe technique was utilized to evaluate item clarity and to facilitate a three‐point rating system (“Essential item,” “Essential but needs revision,” and “Not essential item”). To assess expert feedback, the content validity ratio (CVR) and content validity index (CVI) were calculated for each item. A CVR threshold of ≥0.62 was considered acceptable for item adequacy (Lawshe 1975).

Following expert evaluations, minimal revisions were made, and the revised scale was pilot‐tested with 30 nurses to assess comprehensibility. While modifications were applied to some items based on expert feedback, no items were removed. Nurses who participated in the pilot test were not included in the main sample.

#### 3.2.3 Evaluation of the Psychometric Properties of the Developed Scale

IBM SPSS 25.0 and IBM AMOS 24.0 software packages were used for data analysis. The normality of the data distribution was assessed using skewness and kurtosis values, where values within the range of ±1 were considered to indicate a normal distribution (Hair et al. 2014). CVI of the overall scale and the CVR for each item were calculated based on expert ratings using the Lawshe technique. EFA and CFA were conducted to assess construct validity. A statistical significance level of *p* < 0.05 was adopted. The reliability of the scale was evaluated using the test–retest method and Cronbach's alpha coefficient.

### 3.3 Validity Analysis

To assess the construct validity of the scale, EFA and CFA were performed using Promax rotation in SPSS. The dataset was randomly divided into two subsamples for EFA and CFA, following the random split‐half method (Orçan 2018). The suitability of the dataset for EFA was determined using the Kaiser–Meyer–Olkin (KMO) coefficient (KMO ≥ 0.60) and Bartlett's test of sphericity (*p* < 0.05). Factor retention was based on factor loadings ≥ 0.50 and eigenvalues ≥ 1.0, while items below these thresholds were excluded (Tabachnick et al. 2013).

To evaluate the fit of the factor structure to the data, CFA was conducted in IBM AMOS using the maximum likelihood estimation method. Model‐data fit was assessed using various fit indices, including the normed chi‐square index (χ^2^/df), standardized root mean square residual (SRMR), comparative fit index (CFI), root mean square error of approximation (RMSEA), goodness‐of‐fit index (GFI), incremental fit index (IFI), adjusted GFI (AGFI), Tucker–Lewis index (TLI), and normed fit index (NFI) (Hair et al. 2014).

### 3.4 Reliability Analysis

To assess the internal consistency of the scale, Cronbach's alpha coefficient, item–total correlation coefficients, and inter‐item correlations were analyzed. A Cronbach's alpha value ≥ 0.70 and positive item‐total correlation coefficients > 0.20 were considered acceptable (Hair et al. 2014).

The discriminative power of the items was evaluated by comparing the top and bottom 27% groups, while the temporal stability of the scale was assessed using the test–retest method. The test–retest reliability was analyzed using Pearson's correlation coefficient, with the second administration conducted four weeks after the initial test. Based on Pearson's correlation coefficient, values were interpreted as follows: 0.00–0.10: negligible correlation, 0.10–0.39: weak correlation, 0.40–0.69: moderate correlation, 0.70–0.89: strong correlation, and 0.90–1.00: very strong correlation (Schober et al. 2018).

### 3.5 Data Collection

The data were collected between January and September 2024. The data collection tools were designed using Google Forms. Initially, the researchers shared the study invitation link with volunteer nurses working in public institutions via relevant social media platforms (e.g., Facebook, WhatsApp, and Telegram nursing groups). After completing the form, participants were encouraged to share the invitation link with their colleagues.

When employing snowball sampling, ensuring the continued expansion of participant recruitment is crucial (Leighton et al. 2021). Therefore, throughout the data collection process, the researchers systematically re‐shared the study invitation link every two to three weeks to maintain participant engagement.

### 3.6 Ethical Considerations

Ethical approval was obtained from the Ethics Committee of Gazi University (decision number 22; dated December 27, 2022). The study was conducted in accordance with the ethical principles outlined in the Declaration of Helsinki (World Medical Association 2013). Prior to participation, informed consent was obtained from all nurses via an online form, which clearly explained the purpose of the study, data collection procedures, and participants’ rights. All collected data were anonymized and handled with strict confidentiality and data security protocols. No personal identifiers were recorded. The data were stored in an encrypted digital environment accessible only to the researchers. They were used exclusively for scientific purposes and were not shared with third parties. After completion of the study, the data would be retained for a duration defined by ethical guidelines and then securely destroyed.

## 4 Results

### 4.1 Nurses’ Characteristics

The participants had a mean age of 29.7 years, with 79.2% being female and 20.8% male. Of the participants, 33.9% worked in inpatient wards, and 32.7% worked in intensive care units. Additionally, 72% held a bachelor's degree, and 28% had more than 10 years of work experience. Furthermore, 68.5% had previous experience caring for incarcerated patients (Table 1).

**TABLE 1:** Descriptive characteristics of the nurses (*n* = 336).

Characteristics	Categories	*n*	%
Age (mean ± SD)	29.7 ± 6.8		
Gender	Female	266	79.2
	Male	70	20.8
Department	Inpatient unit	114	33.9
	Intensive care	110	32.7
	Operating room	22	6.5
	Emergency	16	4.8
	Policlinic	18	5.4
	Other^*^	56	16.7
Education level	High school	14	4.2
	Associate degree	16	4.8
	Bachelor's degree	262	72.0
	Graduate degree (Master's/Doctorate)	64	19.0
Years of nursing experience	Less than 1 year	92	27.4
	1–5 years	92	27.4
	6–10 years	58	17.3
	More than 10 years	94	28.0
Experience in caring for prisoners as patients	Yes	230	68.5
	No	106	31.5

* Other: Maternity ward, nursing services administration, blood bank, laboratory, primary health care services, hemodialysis unit.

### 4.2 Findings Regarding the Validity of the Scale

In this study, based on the number of expert evaluations, a minimum CVR threshold of ≥ 0.62, as recommended in the Lawshe technique, was applied. The calculations revealed that the CVR values for each item ranged between 0.80 and 1.00. The overall CVI for the scale was determined to be 0.97. Since CVI > 0.62, the measurements can be considered statistically appropriate.

### 4.3 Factor Analysis

For the first subsample, the absolute skewness values ranged from −0.040 to 0.133, while the kurtosis values varied from −0.424 to 0.265. In the second subsample, skewness values ranged from −0.090 to 0.140, and kurtosis values ranged from 0.374 to 0.280. These results indicate that the normality assumption is met (Hair et al. 2014; Tabachnick et al. 2013).

### 4.4 Exploratory Factor Analysis

To assess the suitability of the collected data for factor analysis, the KMO test was conducted, yielding a KMO value of 0.88. Additionally, Bartlett's test of sphericity was performed to examine the multivariate normality of the dataset and its statistical significance, resulting in χ^2^ = 7009.849, *p* < 0.001. These findings indicate that the collected data are appropriate for conducting factor analysis within the scope of the study.

As a result of the initial EFA, the final version of the scale was formed by removing items that did not load onto any factor or exhibited cross‐loadings. The analysis indicated that the remaining items were grouped into three factors with eigenvalues greater than 1.

A factor loading of at least 0.40 is required for an item to be included in a factor (Stevens 2002). However, Hair et al. (2014) emphasize that factor loadings between 0.30 and 0.40 meet the minimum threshold for interpretation, while values ≥ 0.50 are considered practically significant. Furthermore, items with factor loadings above 0.70 indicate a well‐defined structure. In this study, factor loadings ranged from 0.69 to 0.81 for items in the first factor, from 0.61 to 0.85 in the second factor, and from 0.70 to 0.83 in the third factor (Table 2).

**TABLE 2:** Factor loading and common factor variance.

Subdimensions	Item no.	Factor 1^*^	Factor 2^*^	Factor 3^*^	Common factor variance
Discriminatory attitudes	5	0.819			0.702
	15	0.808			0.710
	7	0.793			0.751
	10	0.777			0.607
	12	0.695			0.602
Emotional discomfort	17		0.853		0.834
	19		0.810		0.700
	16		0.807		0.769
	22		0.617		0.443
Patient equality	9			0.839	0.750
	8			0.753	0.605
	3			0.706	0.648
Eigenvalues		4.692	2.125	1.305	
Percentage of the variance		39.097	17.708	10.878	
Total variance explained		67.683			

* Factor loadings below 0.50 are not displayed in the table.

The construct validity of the scale was assessed by examining the correlation between the total score and subdimensions after the EFA. The correlation coefficients, which range between −1 and +1, indicate the strength of the relationship between variables (Salkind 2017). The findings demonstrated that the correlation coefficients between the total score and the subdimensions ranged from *r* = 0.71 to *r* = 0.79, while the correlations among subdimensions varied between *r* = 0.27 and *r* = 0.49. Specifically, discriminatory attitudes exhibited a moderate correlation with patient equality (*r* = 0.49, *p* < 0.05) and a strong correlation with the total score (*r* = 0.79, *p* < 0.05). Emotional discomfort showed a weak correlation with discriminatory attitudes (*r* = 0.27, *p* < 0.05) and a moderate correlation with patient equality (*r* = 0.31, *p* < 0.05), while it had a strong correlation with the total score (*r* = 0.74, *p* < 0.05). Furthermore, patient equality demonstrated a strong correlation with the total score (*r* = 0.71, *p* < 0.05). These results indicate that all subdimensions are positively and significantly correlated with each other and with the total scale score, supporting the notion that they measure the same underlying construct.

### 4.5 CFA

**FIGURE 2:**
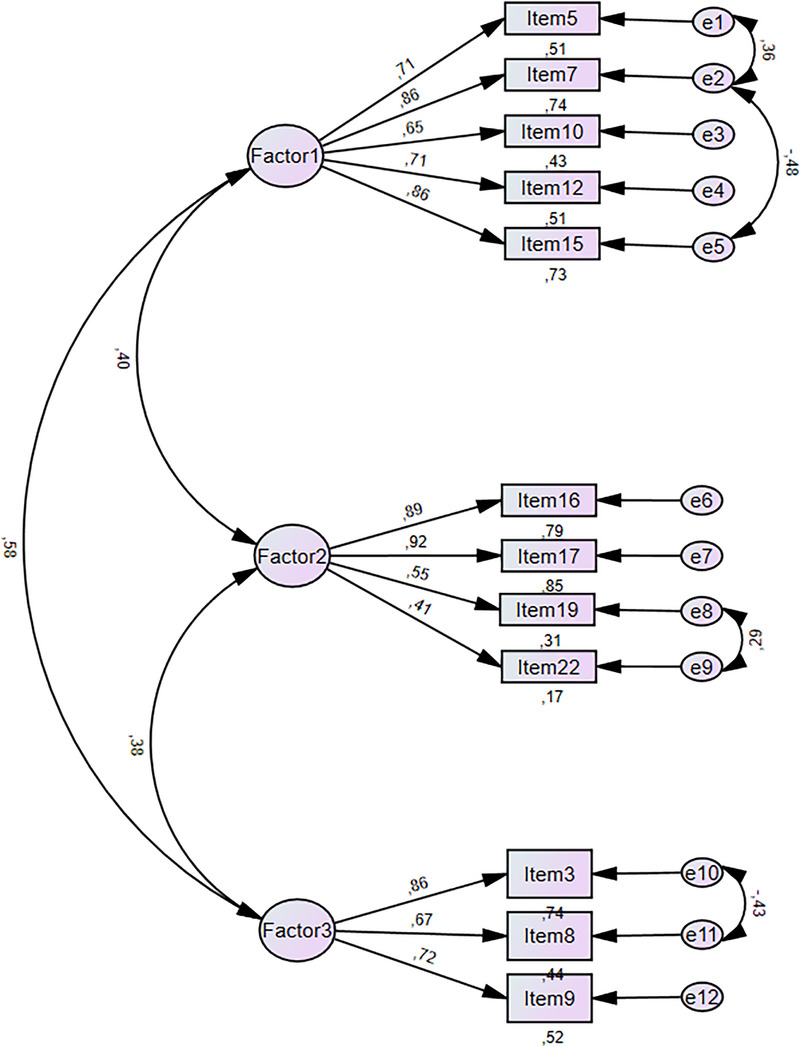
CFA: Standardized scores for the three‐factor structure of the scale.

In this study, CFA was conducted to test whether the factor structure of the developed scale was consistent with that of the original scale (Figure 2).

To evaluate the validity of the model, commonly used fit indices, including χ^2^/df, RMSEA, NNFI, CFI, IFI, NFI, GFI, AGFI, and SRMR, were utilized (Schumacker and Lomax 2004). Following the modification suggestions obtained from CFA to provide evidence for construct validity, the resulting GFIs and their reference values are presented in Table 3.

**TABLE 3:** Goodness‐of‐fit indices used in the research and acceptable value range.

Indexes	Good fit	Acceptable fit	CFA results
χ^2^/df	0 ≤ χ^2^/df ≤ 3	3 ≤ χ^2^/df ≤ 4	3.6 (*p* < 0.001)
AGFI	0.90 ≤ AGFI ≤ 1	0.85 ≤ AGFI ≤ 0.90	0.86
GFI	0.95 ≤ GFI ≤ 1	0.90 ≤ GFI ≤ 0.95	0.92
TLI	0.95 ≤ TLI ≤ 1	0.90 ≤ TLI ≤ 0.95	0.90
IFI	0.95 ≤ IFI ≤ 1	0.90 ≤ IFI ≤ 0.95	0.93
CFI	0.95 ≤ CFI ≤ 1	0.90 ≤ CFI ≤ 0.95	0.93
NFI	0.95 ≤ NFI ≤ 1	0.90 ≤ NFI ≤ 0.95	0.91
RMSEA	0 ≤ RMSEA ≤ 0.05	0.05 ≤ RMSEA ≤ 0.08	0.08
SRMR	0 ≤ SRMR ≤ 0.08	0.05 ≤ SRMR ≤ 0.10	0.06

As a result of the CFA conducted for this scale development study, the statistical significance of each item's standardized analysis results was evaluated using *t*‐values. The *t*‐values ranged from 7.55 to 17.92, and all items were found to be statistically significant at the *p* < 0.001 level. Furthermore, an examination of the fit index values obtained after CFA indicated that the 12‐item measurement tool demonstrated good and acceptable fit indices, confirming its applicability.

### 4.6 Reliability Analysis

One of the most commonly used criteria for assessing scale reliability is Cronbach's alpha, which measures internal consistency. In this study, the reliability analysis of the Nurses’ Attitudes Toward Incarcerated Patients Scale was conducted using the following methods: internal consistency (Cronbach's alpha and item–total correlation), upper–lower 27% group comparison, and test–retest reliability for temporal stability.

In the scale reliability analysis, Cronbach's alpha coefficients were calculated to assess internal consistency. The Cronbach's alpha coefficient for the scale was found to be 0.84, which exceeds the commonly accepted threshold of 0.70 (Hair et al. 2014) (Table 4). To determine whether each item of the developed scale had sufficient discriminative properties in relation to the scale scores, an independent samples *t*‐test was conducted by comparing the upper and lower 27% groups (Table 5).

**TABLE 4:** Reliability of the scale and its subdimensions.

Subdimensions	Item no.	Item–total correlation	Cronbach's alpha if item deleted	Cronbach's alpha
Discriminatory attitudes	5	0.49	0.83	0.87
	7	0.64	0.82	
	10	0.46	0.83	
	12	0.61	0.82	
	15	0.60	0.82	
Emotional discomfort	16	0.62	0.82	0.80
	17	0.60	0.82	
	19	0.30	0.84	
	22	0.35	0.84	
Patient equality	3	0.57	0.82	0.74
	8	0.42	0.83	
	9	0.47	0.83	

**TABLE 5:** Item analysis of the scale *t* values for upper and lower groups of 27%.

Subdimension	Item no.	Group	Mean	SD	df	*t*
Discriminatory attitudes	5	Lower	3.59	1.08	180	10.950^*^
		Upper	4.89	0.31		
	7	Lower	3.38	0.92	180	14.315^*^
		Upper	4.86	0.34		
	10	Lower	4.04	0.96	180	8.230^*^
		Upper	4.91	0.28		
	12	Lower	3.21	1.13	180	12.794^*^
		Upper	4.82	0.38		
	15	Lower	3.40	0.82	180	14.980^*^
		Upper	4.83	0.37		
Emotional discomfort	16	Lower	1.94	0.88	180	14.283^*^
		Upper	3.98	1.03		
	17	Lower	2.16	0.96	180	13.810
		Upper	4.09	0.91		
	19	Lower	2.53	1.00	180	6.763
		Upper	3.62	1.16		
	22	Lower	2.85	0.96	180	5.923
		Upper	3.82	1.22		
Patient equality	3	Lower	3.39	0.80	180	12.771^*^
		Upper	4.65	0.49		
	8	Lower	3.41	1.05	180	9.264^*^
		Upper	4.62	0.66		
	9	Lower	3.53	0.79	180	9.977^*^
		Upper	4.64	0.70		

* *p* < 0.05.

### 4.7 Test–Retest

To assess the temporal stability of the scale, the test–retest method was applied, and the scale items were re‐administered to nurses four weeks later. The test–retest results were as follows: Discriminatory attitudes subdimension: *r* = 0.575, *p* < 0.05; emotional discomfort subdimension: *r* = 0.429, *p* < 0.05; patient equality: *r* = 0.600, *p* < 0.001; total score: *r* = 0.542, *p* < 0.05. A statistically significant positive correlation was found between the test–retest total scores of the scale (*p* < 0.05), indicating the stability of the scale over time.

## 5 Discussion

The present study evaluated the psychometric properties and validity analysis of the scale developed to assess nurses’ attitudes toward incarcerated patients. The findings indicated that the scale meets the validity and reliability criteria outlined in the literature (Carpenter 2017). The items in the developed scale reflect opinions related to attitudes and measure the level of attitudes. In this context, the scale is expected to contribute to the literature by providing a valid tool for assessing nurses’ attitudes toward incarcerated patients.

The KMO test is a statistical measure used to determine whether the dataset is suitable for factor analysis. A KMO value between 0.80 and 0.90 indicates that the scale demonstrates a strong performance. In this study, the KMO value was found to be 0.88. Additionally, the Bartlett's test of sphericity was found to be statistically significant (*p* < 0.001). These results indicate that the data are appropriate for factor analysis.

In multifactorial designs, explaining 40%–60% of the variance is generally considered sufficient for construct validity (Field 2018). Since there is no previous study assessing nurses’ attitudes toward incarcerated patients, a comparison was made with similar studies. For instance, in the Criminal Perceptions Scale developed by Gönültaş et al. (2019), the total variance explained was 52.4%. Similarly, in the Attitudes Toward Former Offenders Scale by Batur and Akbaş (2023), the total variance explained was 53.2%, while in the Attitudes Toward Offenders Scale by Pereyra and Moreno (2001), it was 55%. Moreover, in the Nurses' Attitudes Toward Forensic Psychiatric Patients Scale by Baysan‐Arabacı and Çam (2011), the total variance explained was 44%.

In this study, the three‐factor structure obtained from the EFA accounted for 67.6% of the total variance. The first factor, which primarily emphasizes discriminatory attitudes, was labeled “Discriminatory Attitudes” and accounted for 39% of the variance. The second factor, explaining 17.7% of the variance, primarily includes emotional reactions and attitudes and was named “Emotional Discomfort.” The third factor, which explains 10.8% of the variance, was labeled “Patient Equality.” To assess the relationship between subdimensions, correlations among factors were calculated. The results indicated a moderate correlation (0.49) between the discriminatory attitudes and patient equality factors, a weak correlation (0.27) between the discriminatory attitudes and emotional discomfort factors, and a weak but statistically significant correlation (0.31) between emotional discomfort and patient equality factors. These findings support the validity of the developed scale.

In the second phase of the study, CFA was conducted to validate the structure identified by EFA. Before implementing the suggested modifications, the fit index values were as follows: χ^2^/df = 5.11 (*p* < 0.001), RMSEA = 0.11, CFI = 0.89, AGFI = 0.82, IFI = 0.89, GFI = 0.88, NFI = 0.87, TLI = 0.86. Four modification suggestions emerged concerning the relationships between the following items: M5 and M7, M7 and M15, M19 and M22, and M3 and M8. These modifications were considered based on the assumption that they would contribute to model improvement. After incorporating the suggested modifications, the updated fit index values were as follows: χ^2^/df = 3.6 (*p* < 0.001), RMSEA = 0.08, AGFI = 0.86, IFI = 0.93, CFI = 0.93, NFI = 0.91, TLI = 0.90, GFI = 0.92, SRMR = 0.06. These results indicate that the 12‐item measurement tool has good and acceptable fit indices, suggesting that it is applicable and valid (Schumacker and Lomax 2004). Furthermore, the analysis revealed that all item *t*‐values exceeded the 1.96 criterion, indicating that the items had a statistically significant relationship with their respective factors (Kline 2023).

In the current study, the Cronbach's alpha coefficient of the scale was determined to be 0.84. This result is consistent with similar scales in the literature. For instance, the Attitudes Toward Prisoners Scale developed by Melvin et al. (1985) was assessed for internal consistency using split‐half reliability analysis, yielding reliability values ranging between 0.84 and 0.92. Similarly, in the Attitudes Toward Self‐Harming Prisoners Scale by Garbutt and Casey (2015), the Cronbach's alpha coefficient was reported as 0.72. Additionally, the Attitudes Toward Former Prisoners Scale by Batur and Akbaş (2023) had a Cronbach's alpha of 0.88, while the Attitudes of Nurses Toward Forensic Psychiatric Patients Scale by Baysan‐Arabacı and Çam (2011) reported a value of 0.86. Furthermore, the Perceptions of Criminals Scale by Gönültaş et al. (2019) had an alpha coefficient of 0.82, and the Attitudes Toward Criminals Scale by Pereyra and Moreno (2001) reported values ranging between 0.56 and 0.79. The Cronbach's alpha coefficient obtained in our study aligns with these findings, indicating that the scale is a reliable measurement tool.

To determine the accuracy of the three‐dimensional, 12‐item structure of the measurement tool in assessing the intended characteristics, an item analysis was conducted. The obtained item–total correlation values ranged between 0.30 and 0.64. Item–total correlation values equal to or greater than 0.30 are considered evidence supporting the validity of the items in the scale (Hair et al. 2014). These findings indicate that the item–total test correlation coefficients of the scale exhibit a weak to moderate relationship, demonstrating that each item effectively measures the targeted construct.

The analysis of *t*‐values between the upper and lower 27% groups for the scale's item scores indicates that the scale possesses the ability to differentiate between individuals who exhibit the targeted characteristic and those who do not. In other words, it effectively distinguishes individual differences (Cohen and Swerdlik 2018). In the present study, the *t*‐test values were found to be statistically significant, confirming that the scale demonstrates discriminative validity.

To ensure the test–retest reliability of the measurement instrument, it is expected to yield consistent results over time (Cohen and Swerdlik 2018). In this context, the correlation analyses conducted to assess the stability of the scale over time demonstrated moderate, positive, and statistically significant correlations across all subdimensions and the total scale score. These findings indicate that the scale exhibits test–retest reliability both overall and across each subdimension.

### 5.1 Strengths and Limitations

The most significant contribution of this study to the literature is the development of a scale measuring nurses' attitudes toward incarcerated patients. While existing literature includes several scales assessing attitudes toward offenders or detainees, these instruments primarily focus on the general public's perceptions. This newly developed scale provides a valuable opportunity to assess nurses' attitudes and examine their impact on healthcare delivery.

This study has certain limitations. First, the “Nurses' Attitudes Toward Incarcerated Patients Scale” was tested exclusively in Türkiye. As a result, the generalizability of the scale may be limited for nurses in different geographic regions or culturally diverse populations. Additionally, the validity and reliability analyses of the scale were conducted solely based on the data obtained in this study. Lastly, since the data collection method relied on self‐reported surveys, potential sources of bias, such as social desirability bias, may have influenced participants’ responses.

## 6 CONCLUSION

The findings indicate that the developed scale is a valid and reliable measurement tool in terms of both validity and reliability. The results of EFA and CFA confirmed that the scale has a three‐factor structure, and the correlations between the factors were found to be statistically significant. Furthermore, the Cronbach's alpha coefficient of 0.84 supports the internal consistency of the scale, demonstrating its acceptable reliability.

For future studies, it is recommended that the validity and reliability analyses of the scale be conducted in different cultural and geographical populations. Additionally, a comprehensive examination of the factors influencing nurses’ attitudes toward incarcerated patients is suggested.

This study highlights the need for policies that address nurses’ attitudes toward incarcerated patients. The developed scale can help identify biases and emotional discomfort, guiding targeted training and institutional interventions. Integrating this tool into nursing education and practice may promote equitable, ethical, and unbiased healthcare delivery for incarcerated individuals.

## Author Contributions

Conceptualization: BDG, SAA, and AK. Data curation: BDG and NKK. Methodology: BDG, SAA, NKK, and AK. Visualization: BDG, SAA, NKK, and AK. Validation: BDG, SAA, NKK, and AK. Formal analysis: BDG and AK. Investigation: BDG, SAA, NKK, and AK. Writing—original draft: BDG, SAA, NKK, and AK. Project administration: SAA. Supervision: SAA and AK.

## Conflicts of Interest

The authors declare no conflict of interest regarding the development, validation, or investigation of the tool in this study.
